# HIV Nuclear Entry: Clearing the Fog

**DOI:** 10.3390/v2051190

**Published:** 2010-05-11

**Authors:** Vaibhav B. Shah, Christopher Aiken

**Affiliations:** Department of Microbiology and Immunology, Vanderbilt University School of Medicine, A-5301 Medical Center North, Nashville TN 37232-2363, USA; E-Mail: vaibhav.b.shah@vanderbilt.edu

**Keywords:** HIV-1, capsid, nuclear entry, TNPO3, nucleoporins

## Abstract

HIV-1 and other lentiviruses have the unusual capability of infecting nondividing cells, but the mechanism by which they cross an intact nuclear membrane is mysterious. Recent work, including a new study (Lee, K.; Ambrose, Z.; Martin, T.D.; Oztop, I.; Mulky, A.; Julias, J.G.; Vandergraaff, N.; Baumann, J.G.; Wang, R.; Yuen, W. *et al*. Flexible use of nuclear import pathways by HIV-1. *Cell Host Microbe* **2010**, *7,* 221–233) confirms that the viral capsid plays a key role in HIV-1 nuclear entry in both dividing and nondividing cells. The identification of mutations in the viral capsid that alter the virus’s dependence on host cell nucleoporins represents an important advance in this poorly understood stage of the virus life cycle.

It has been recognized for nearly two decades that among retroviruses, HIV-1 and other lentiviruses can efficiently infect nondividing as well as dividing cells. Nondividing cells that have been often characterized include terminally differentiated macrophages—important targets of HIV-1 infection *in vivo*—and laboratory cell lines that have been arrested in the cell cycle upon treatment with chemical inhibitors or radiation. Most other retroviruses lack the ability to infect such cells, presumably relying on mitosis and the accompanying breakdown of the nuclear envelope to gain access to the chromatin for integration. This important difference between lentiviruses and other retroviruses has naturally prompted studies aimed at identifying the viral and host factors that mediate lentiviral infection of nondividing cells (reviewed in [1]). Over the years, the field has chased a plethora of viral determinants for infection of nondividing cells, and by inference, traversal through the nuclear pore. After investigations of the viral MA, Vpr, and IN proteins apparently failed to withstand the test of time [2–6], a triple stranded viral DNA structure known as the flap was implicated [7]. Later evidence appears to have excluded all of these candidates and implicated a new viral factor—the capsid—in HIV-1 nuclear import [8,9].

The latest episode in this saga was recently provided by KyeongEun Lee and coworkers and reported in *Cell Host and Microbe* [10]. By screening a library of mouse cDNAs for clones that would render cells resistant to HIV-1, the authors identified a form of the RNA cleavage polyadenylation and specificity factor CPSF6. The resulting truncated protein, CPSF6-358, is not an endogenously expressed splice variant of CPSF6, but resulted from random cDNA priming of the library. While CPSF6 is normally enriched in the nucleus, CPSF6-358 was predominantly cytoplasmic. The antiviral effect of CPSF6-358 was also observed in primary CD4^+^ T cells and was also effective against HIV-2 and SIV, but did not inhibit FIV or MLV. CPSF6-358 inhibited HIV-1 at an early postentry step in infection, a block that the authors narrowed down to the nuclear import step. However, the full-length CPSF6 protein had only a slight inhibitory effect on HIV-1 infection. While some researchers might have chalked up the result to an artifact of the cloning/selection procedure and dropped the project, the authors persevered by selecting for a viral mutant that had acquired resistance to CPSF6-358 (the inhibitory protein). They identified a single point mutation in the viral capsid protein (CA) that allowed efficient infection in the presence of CPSF6-358. Previous studies from the Emerman group had shown that CA is a key requirement for HIV-1 infection of nondividing cells [8], and point mutations in CA can selectively impair this function [11]. Indeed, Lee and coworkers found that CPSF6-358, like some CA mutations, was much more inhibitory when the cells were arrested in the cell cycle by treatment with the DNA synthesis inhibitor aphidicolin. The CA mutation N74D relieved the block in arrested as well as dividing cells, demonstrating that the mutant virus is generally resistant to the inhibitory protein.

The authors next turned to recent observations emerging from genome-wide RNAi screening that identified specific host proteins in HIV-1 infection. Several of these, including TNPO3 and several nucleoporins (NUPs), play important roles in cellular protein nuclear import [12]. The authors asked an insightful question: does the N74D mutation alter HIV-1 dependence on nuclear import factors? The answer was surprising: infection by HIV-1 bearing the N74D substitution in CA was relatively resistant to depletion of TNPO3 in the target cell. A collaborative study with the Engelman group recently identified CA as the principal determinant of HIV-1 dependence on TNPO3 [13], even though TNPO3 was previously shown to bind to the viral integrase (IN) protein [14]. In actuality, it was the former result, presented nearly two years prior at the Cold Spring Harbor Retroviruses meeting, which prompted the hypothesis that CA governs HIV-1 dependence on TNPO3. Lee and coworkers also found that besides N74D, other CA mutants, including two previously shown to be impaired for infection of nondividing cells [11], were less dependent on TNPO3. The N74D mutation also reduced the virus’s dependence on RANBP2 and NUP153 but not other nucleoporins including NUP155 and NUP160. The mutant was actually hypersensitive to depletion of NUP85 and NUP155, thus mimicking the requirements for FIV infection and leading the authors to conclude that HIV-1 can utilize various host factors for gaining access to the nucleus—*i.e.*, if access to one NUP is blocked, a dependence on others is revealed. TNPO3 is not required for infection by murine leukemia virus (MLV), a retrovirus that lacks the ability to infect nondividing cells and is thought to undergo a slow uncoating process. However, a role for NUPs in MLV infection remains to be tested. Overall, the results clearly reinforce the role of CA as a determinant in HIV-1 nuclear entry and demonstrate that CA controls the virus’s interactions with host factors.

How exactly does CPSF6-358 block nuclear entry of HIV-1? Answering this will require additional investigations. Nonetheless, Lee *et al.* showed that the protein binds to tubular complexes composed of a fragment of the HIV-1 Gag protein *in vitro*, and the N74D mutation in CA reduces the binding. Logically, the authors propose that binding of CPSF6-358 to the viral capsid blocks the engagement of host factors required for uncoating and/or nuclear entry. The putative factor could be TNPO3 or another importer. In this respect, CPSF6-358 inhibition would appear to mimic the action of the rhesus macaque TRIM5α, which targets CA and blocks HIV-1 nuclear entry in cells in which proteasome activity has been blocked with chemical inhibitors [15]. The authors’ model is consistent with their observation that the HIV-1 CA mutants E45A and Q63A/Q67A, which were resistant to CPSF6-358 in dividing cells where HIV-1 may passively enter the nucleus, were inhibited by more than 200-fold in arrested cells. Such mutants may be even more dependent on specific host factors owing to putative defects in interactions with others.

As with all novel and interesting research, the work of Lee *et al.* raises new questions, particularly regarding the relationship between retroviral capsid function and nuclear import. First, why are TNPO3 and NUPs important for lentiviral infection of cycling cells—in which nuclear import is presumably passive? Surprisingly, arrest of Hela cells with aphidicolin did not lead to an enhanced TNPO3 dependence, yet CPSF6-358 restriction was potentiated in arrested cells. Thus, some blocks to nuclear entry appear general, while others are more selective for nondividing cells. Unfortunately, aphidicolin was toxic to cells depleted of NUPs, thus precluding a more detailed analysis of the requirement for NUPs in nondividing cells. Second, what role does uncoating—defined as shedding of the viral capsid—play in viral nuclear entry? Evidence for HIV-1 uncoating following docking to the nuclear pore has been reported [16]. Several CA mutations that confer cell cycle dependence to HIV-1 infection appear to delay HIV-1 uncoating, suggesting that nuclear entry is dependent on uncoating [11,17,18]. It is possible that CPSF6-358, by engaging the viral capsid, inhibits uncoating thus restricting passage of the viral preintegration complex through the nuclear pore in nondividing cells due to size or geometric limitations. In dividing cells, persistent association of the capsid may also confer a block to viral integration. It also seems possible that CPSF6-358 directly inhibits circularization of the proviral DNA, potentially confounding the analysis of nuclear import defects. Further studies of host functions controlling early stages of the HIV-1 life cycle will undoubtedly reveal novel details of the viral-host Pas de deux.
